# Comparison of Open-Source Reverse Vaccinology Programs for Bacterial Vaccine Antigen Discovery

**DOI:** 10.3389/fimmu.2019.00113

**Published:** 2019-02-14

**Authors:** Mattia Dalsass, Alessandro Brozzi, Duccio Medini, Rino Rappuoli

**Affiliations:** ^1^GlaxoSmithKline, Siena, Italy; ^2^Dipartimento di Scienze Cliniche e Biologiche, Università degli Studi di Torino, Turin, Italy

**Keywords:** reverse vaccinology (RV) programs, antigen, bacterial pathogens, potential vaccine candidates (PVCs), bacterial protective antigens (BPAs)

## Abstract

Reverse Vaccinology (RV) is a widely used approach to identify potential vaccine candidates (PVCs) by screening the proteome of a pathogen through computational analyses. Since its first application in Group B *meningococcus* (MenB) vaccine in early 1990's, several software programs have been developed implementing different flavors of the first RV protocol. However, there has been no comprehensive review to date on these different RV tools. We have compared six of these applications designed for bacterial vaccines (NERVE, Vaxign, VaxiJen, Jenner-predict, Bowman-Heinson, and VacSol) against a set of 11 pathogens for which a curated list of known bacterial protective antigens (BPAs) was available. We present results on: (1) the comparison of criteria and programs used for the selection of PVCs (2) computational runtime and (3) performances in terms of fraction of proteome identified as PVC, fraction and enrichment of BPA identified in the set of PVCs. This review demonstrates that none of the programs was able to recall 100% of the tested set of BPAs and that the output lists of proteins are in poor agreement suggesting in the process of prioritize vaccine candidates not to rely on a single RV tool response. Singularly the best balance in terms of fraction of a proteome predicted as good candidate and recall of BPAs has been observed by the machine-learning approach proposed by Bowman (1) and enhanced by Heinson (2). Even though more performing than the other approaches it shows the disadvantage of limited accessibility to non-experts users and strong dependence between results and *a-priori* training dataset composition. In conclusion we believe that to significantly enhance the performances of next RV methods further studies should focus on the enhancement of accuracy of the existing protein annotation tools and should leverage on the assets of machine-learning techniques applied to biological datasets expanded also through the incorporation and curation of bacterial proteins characterized by negative experimental results.

## Introduction

Reverse Vaccinology (RV) is a genome-based approach developed for the first time in early 1990's by Rappuoli (3) to identify meningococcal protein vaccine candidates in Group B *meningococcus* (MenB). In its original conception, since antigens inducing humoral antibody response are primarily located in extracellular or outer membrane district, all the open reading frames extracted from the genome sequence of MenB strain MC58 were screened to select proteins predicted to be surface exposed, secreted or lipoproteins.

**Figure 1:**
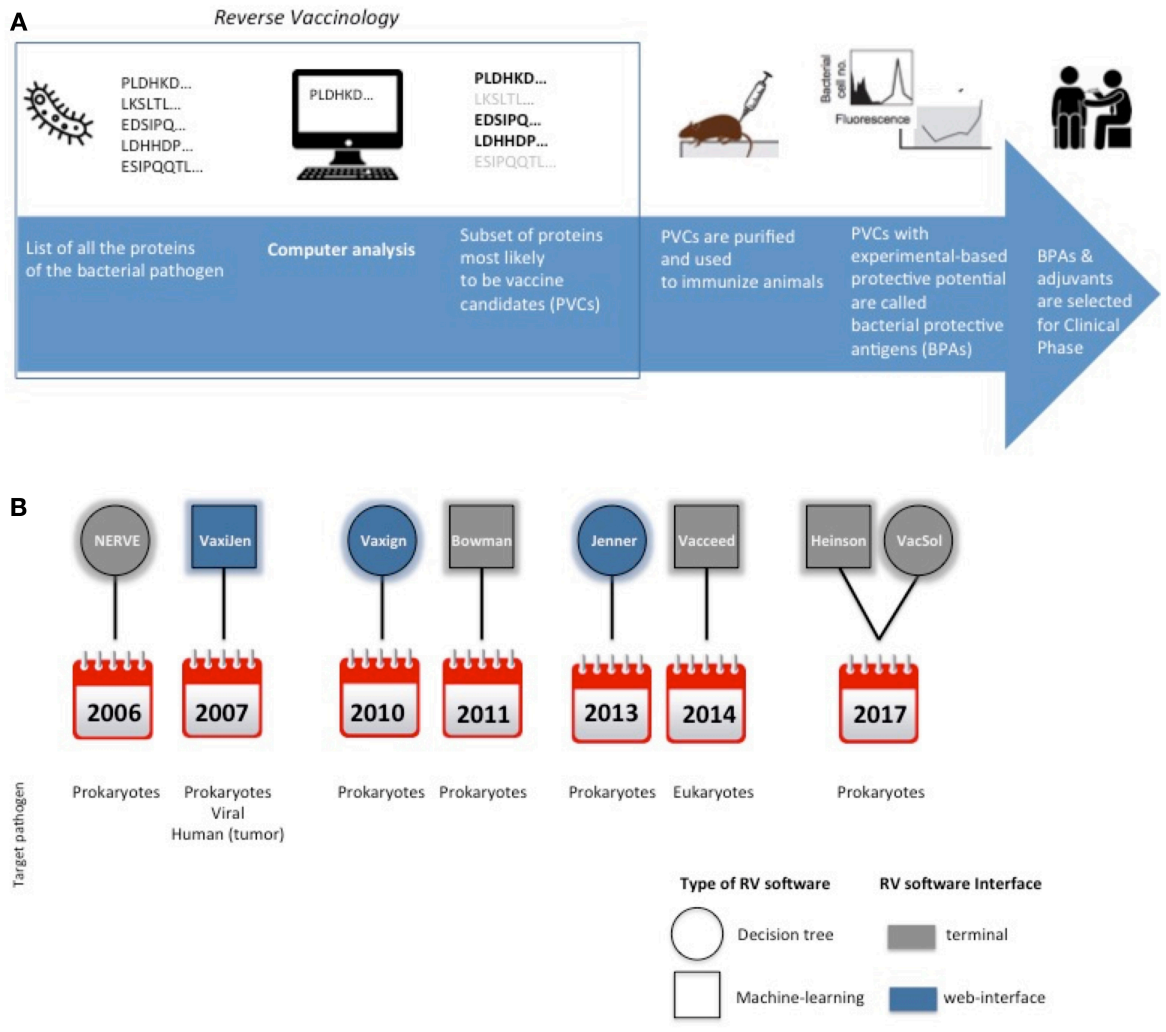
Cartoon schematically representing the main steps for protein subunit vaccines development. In the square is highlighted the Reverse Vaccinology part **(A)**. Timeline of the delivery of RV standalone programs and their main characteristics in terms of type of software, interface and target pathogen **(B)**.

RV approach has revolutionized vaccine development by adopting computerized screening of protein sequences from the pathogen as the first step of the process, to select a subset of promising antigens, aka potential vaccine candidates (PVCs) (Figure 1A).

RV offers two main advantages compared to traditional vaccine development approaches: (i) identification of candidate antigens without the need to grow the pathogen (ii) identification of any antigen independently by its purified quantity to be suitable for vaccine testing.

Proteins returned by RV methods are called throughout this review PVCs (Potential Vaccine Candidates). Other names given to the selected proteins are VCs (Vaccine Candidates), VTs (Vaccine Targets), PVCs (Protein Vaccine Candidates). PVCs identified by RV undergo *in-vitro* and *in-vivo* validation through experimental assays aimed at confirming their protective potential. Each pathogen has its specific experimental assays and it is hard to standardize a common set of experimental features; the most common experimental evidences are the protection outcomes in animal models against virulent bacterial challenge or results obtained from correlate to protection like the human bactericidal assay (4). In the context of this review we refer to any candidate protein that gave positive results in confirmatory preclinical experimental assays as BPAs (bacterial protective antigens). In the literature synonymous of BPAs are protective antigens (PAg), known antigens (KA), or known protective antigens (KPA). Lists of BPAs for different bacteria or viruses might be found in databases like Violin (Protegen) (5) or mining the literature. A comprehensive review of the main biological features characterizing BPAs deposited in Violin (Protegen) might be found in Ong et al. (6).

### The First RV Protocol

The first RV protocol started with the prediction of all open reading frames from the genome of MenB (strain MC58), in total 2,158. These open reading frames were screened to search for homology to bacterial surface-associated proteins using FASTA (7) and PSI-BLAST program (8). Proteins with no hits found (hypothetical proteins) were analyzed by PSORT (9), SignalP (10), and TMPRED program (11) to search for putative lipoproteins, secreted proteins, outer membrane, or periplasmatic proteins.

From the 2,158 proteins, 570 were selected as PVCs. Out of them 350 were successfully expressed in *Escherichia coli* and injected to immunize mice. Sera from immunized animals were screened in a serum bactericidal assay (SBA)—a correlate of protection against invasive meningococcal diseases—and proteins with negative results were discarded. Among the 28 proteins able to induce bactericidal activity, 5 candidates were selected for final formulation and, combined to outer membrane vesicles (OMVs), later approved with the commercial name of *Bexsero*® (12).

### RV Programs Overview

In the following years the RV protocol was successfully applied to other bacterial pathogens. These pathogens include *Chlamydia pneumonia* (13), *Streptococcus pneumoniae* (14) in which open reading frames encoding putative surface proteins and with significant homology to virulence factors of other bacteria were selected and *Porphyromonas gingivalis* (15), in which PVCs were identified by searching for global homology to proteins of known surface exposure or virulence. In these cases, the selection criteria to identify PVCs were restricted to extracellular subcellular localization and to homology to virulence factors already known in other bacterial species. A review about these first applications might be found in Masignani et al. (16).

Only in 2006 the first standalone RV program, distributed with the name of NERVE (New Enhanced reverse Vaccinology Environment), was published (17). Since then several other pathogen-independent RV programs have been released.

Until now there has been no comprehensive review of the available open-source RV programs and a systematic comparison on a benchmark dataset was missing. In this review we compared 6 open-source standalone RV programs designed for bacterial pathogens: NERVE, VaxiJen (18), Vaxign (19), Bowman-Heinson (1, 2), Jenner-predict (20), and VacSol (21). We tested them on 11 different bacterial proteomes.

#### RV Programs Categories

RV packages can be categorized in two types, according to their algorithmic approach: decision-tree or “filtering” and machine learning or “classifying.” Both types take as input protein sequences and call them as PVCs or not-PVCs.

##### Decision-tree or filtering RV programs

They are flowchart-like programs: the pathogen's protein sequences are passed through a series of filters until a subset is identified as PVCs. The filters are done on protein features that can be directly measured, like the molecular weight, or predicted by a computational program, like the subcellular localization or the probability to be an adhesion protein. When the filter is applied on a numerical feature (e.g., number of predicted transmembrane domains) an *a-priori* cut-off is used. Decision tree RV programs differ each other by the number of filters adopted. Examples of decision tree RV tools are NERVE (17), Vaxign (19), Jenner-predict (20), and VacSol (21).

##### Machine-learning or classifying RV programs

These kinds of applications earlier aggregate the features measured or predicted on the pathogens' protein sequences into a matrix and then, given a known set of training examples of PVCs and not-PVCs, an algorithm builds a model that assigns new input proteins to one of the two classes usually in a probabilistic way. Machine-learning RV programs don't discard proteins, like decision tree RV tools do, but rank the entire set of input proteins for their likelihood of being a PVC. This results to be very useful when preclinical confirmatory assays must be planned since the experimenter might begin with the most promising candidates ranked in top positions.

Machine-learning RV tools are newer in the field and better intercept the increasing attention data analytics is paying to artificial intelligence methods. RV machine-learning tools differ each other from the type of classification algorithm they use, from the number of features they measure and from the size and assortment of proteins that constitute the training set.

Examples of machine-learning RV tools are VaxiJen (18), Vacceed (22) -designed for eukaryotes pathogens- and the method described in Bowman et al. (1) and revised by Heinson et al. (2).

#### Programs Interface

The interfaces to the RV programs fall into two categories, those that operate on the command line and those that have a graphical interface.

Command line input allows for high throughput analysis but has a high barrier to entry for non-technical users. Graphical interfaces, such as web-sites, provide point and click interfaces that non-technical users find easier to use initially, however, they are often limited to the analysis of a few samples at a time.

A synoptic summary of the types, year of release and interfaces of the six programs is provided in Figure 1B.

### Software Description

In this section we describe one by one each of the six RV programs object of study of this review. We refer the reader to each specific publication for any further details. Table 1 summarizes the criteria used by each of the six programs to identify PVCs and reports main advantages or disadvantages come upon their usage.

**Table 1:** Synoptic summary of the main characteristics of the six programs tested.

**Tool**	**Category**	**PVC selection criterion**	**Advantage**	**Disadvantage**	**Number of studies that used the tool**
NERVE	Decision-tree	No cytoplasmatic protein < 2 transmembrane helices High adhesin probability No homology with human proteins	Input and output data are automatically structured in a database	Not updated	4
VaxiJen	Machine-learning	Output probability greater than a cut-off (0.5)	Very fast Graphical interface	Fixed training datasets (100 known bacterial antigens, 100 putative non-antigens)	20
Vaxign	Decision-tree	No cytoplasmatic protein < 2 transmembrane helices High adhesin probability No homology with human and mouse proteins	Regularly maintained Easy to use and intuitive	Download of the results is limited to 500 proteins	18
Jenner-predict	Decision-tree	No cytoplasmatic protein < 2 transmembrane helices Presence of Pfam domains involved in host-pathogen interaction and pathogenesis	Upload and download of large datasets	Temporarily unavailable	1
Bowman-Heinson	Machine-learning	Output probability greater than a cut-off (0.5)	Larger training set (200 known bacterial antigens, 200 putative non-antigens)	Annotation tools for eukaryotes used for bacterial proteins Pipeline not delivered	0
VacSol	Decision-tree	No cytoplasmatic protein < 2 transmembrane helices No homology with human proteins Essential gene Virulence factor	User-friendly interface	Too restrictive	0

#### NERVE (17)

NERVE (New Enhanced Reverse Vaccinology Environment) has the primacy to be the first RV standalone software. It is a decision tree and command line tool. Once installed in a Unix-like operating system (NERVE is implemented in Perl programming language), the tool imports the sequences of the pathogen proteins and launches computational programs to predict five biological features:

1. subcellular localization (pSORT) (23)
2. adhesion probability (SPAAN) (24)
3. topology (HMMTOP) (25)
4. sequence similarity with human proteins (BLASTp) (8)
5. conservation in other strain of the same species (BLASTp).

NERVE parses the results of the five programs and stores the results in a MySQL database.

NERVE uses *a priori* cut-offs to select the PVCs. Based on tests done on 10 proteomes (*Bacillus anthracis, Pseudomonas aeruginosa, Yersinia pestis, Streptococcus agalactiae V, III, Ia, Neisseria meningitidis B, Porphyromonas gingivalis, Borrelia burgdorferi, Chlamydia trachomatis D*) the authors of NERVE suggest the following criterion to identify PVCs: any non-cytoplasmatic protein, with no more than 2 predicted transmembrane helices, with a predicted probability of being and adhesin >0.46 or 0.38 and without sequence similarity to human proteins.

NERVE shows the advantage to be very simple and intuitive; it also allows the user to change the filtering cut-offs according to his/her preferences for long or short lists of PVCs.

NERVE has not been updated since its first release: some Perl libraries became obsolete and to be used not negligible changes must be done to the source code. Homology with human proteins is done comparing by BLAST algorithm each pathogen protein sequence against a dataset of potential MCH ligands derived from the database MHCPEP (26) that has not been updated since 1998.

#### VaxiJen (18)

Published soon after NERVE, is the first RV software adopting machine learning strategy. VaxiJen proposes an alignment-independent method for antigen prediction based on auto cross covariance (ACC) transformation of protein sequences into uniform equal-length vectors. Differently by other RV programs, VaxiJen might predict not only bacterial but also viral and tumor antigens. For bacterial antigens prediction VaxiJen applies ACC transformation to a set of 100 known bacterial antigens that the authors derived mining the literature; a protein was included in the set of known bacterial antigens if it (or part of it) was shown to induce a protective response in an appropriate animal model after immunization. Conversely a twin-set of 100 non-antigens was constructed to mirror the antigen set, randomly selecting proteins from the same set of species without similarity to the set of the 100 known antigens (BLAST expectation value of 3.0 was used). Two-class discriminant analysis by partial least squares was applied to the merged set (200 proteins) to derive a model of prediction that the user might apply on his own dataset of proteins uploading a file through a web-interface.

VaxiJen is a web-interface program. The results page reports antigen probability (as a fraction of unity) for each protein. Criterion to call PVCs is any protein with an antigen probability above a threshold (defaults 0.5).

VaxiJen is the only tool currently allowing classification based solely on the physiochemical properties of protein sequences without any related biological or functional information.

While very easy to use and very fast a major limitation is though represented by the fact that, at least in its current release, it is not possible for the user to change the training dataset upon which the prediction model is derived. A review of VaxiJen applications during the last years might be found in Zaharieva et al. (27).

#### Vaxign (19)

Vaxign is decision-tree software that works via web-interface. Vaxign is available in two forms: *Vaxign Query* that provides precomputed results for users to explore, and *Dynamic Vaxign Analysis* that allows dynamic execution and result visualization.

In *Dynamic Vaxign Analysis*, likewise NERVE, it runs different external computational programs on input protein sequences to predict five biological properties:

1. Subcellular localization (pSORT) (23).
2. Number of transmembrane helices (HMMTOP) (25).
3. Adhesin probability (SPAAN) (24).
4. Similarity to host (human, mouse, pig) proteins (OrthoMCL) (28).
5. MHCI and MHCII epitopes binding (Vaxitope) (19).

The authors, analyzing 11 known protective antigens from four bacterial pathogens strains (*N. meningitidis, H. pylori, B. anthracis, M. tuberculosis*), suggest the following criterion to identify PVCs: any protein surface exposed, with no more than one transmembrane helix, with probability to be an adhesin >0.51 and no sequence similarity to any host protein (human and mouse).

Vaxign mostly resembles NERVE in terms of the protein features predicted, computational programs used and thresholds set to call PVCs, though there are differences:

- They both use pSORT to predict subcellular localization, but NERVE parses the original output and in case the probability to be “Cytoplasmatic” is null it reports “Non-cytoplasmatic.” On the contrary Vaxign keeps the original output of Psort.
- NERVE to call a PVC sets two different thresholds at the probability predicted by SPAAN of being an adhesion: 0.46 if the candidate is predicted “not cytoplasmatic” and 0.38 if predicted “extracellular.”
- Vaxign uses OrthoMCL (28) to calculate the homology to host proteins and the conservation on different strains of the pathogen, whereas NERVE implements a BLASTp query against a not updated dataset of MCHI binding epitopes derived from MHCPEP (26). NERVE filters PVCs for sequence similarity only against human proteins while Vaxign allows also against mouse and pig proteins.

#### Jenner-Predict (20)

It is decision-tree software published in 2013. Jenner-predict identifies PVCs by filtering upon:

1. Subcellular localization (pSORT, version 3.0) (29).
2. Number of transmembrane helices (HMMTOP, version 2.0) (25).
3. Presence of Pfam domains involved in host-pathogen interactions and pathogenesis (30).

Pfam domains include classes of adhesion, invasion, toxin, porins, colonization, virulence, flagellin, penicillin-binding, choline-binding, transferring-binding, fibronectin-binding, and solute-binding.

The criterion to identify PVCs for Jenner-predict is: any non-cytosolic protein with <3 trans-membrane helices and with at least one hit in the list of Pfam domains involved in host-pathogen interactions and pathogenesis. This final list of PVCs is then ranked according to the degree of conservation in different pathogenic and non-pathogenic strains, presence of known epitope sequences (both B and T epitopes) and degree of conservation with human proteins.

The novelty of Jenner-predict is to relax the criterion applied by NERVE and Vaxign on adhesin-likeliness to call PVCs. Jenner-predict doesn't use SPAAN to predict the probability for a candidate to be an adhesion but uses Pfam domains.

Differently by Vaxign, Jenner-predict uses the sequence similarity to human proteins only as a score to rank PVC. Jenner-predict at the time of writing is unavailable for users through its web-interface. We contacted directly the authors to ask for a local evaluation of the software on our benchmark dataset.

#### Heinson-Bowman (1, 2)

We called this method with the names of the first authors who published a machine learning RV method initially in 2011 (1) and then enhanced the classifier publishing the results in 2017 (2). Bowman et al. (1) merged the existing tools NERVE and VaxiJen, adopting from NERVE the idea of use a set of protein annotation tools and from VaxiJen the use of a machine-learning classifier.

The method uses a Support Vector Machine (SVM) classifier using a training dataset constituted by 200 bacterial protective antigens (BPA) extracted from literature. Bacterial protective antigens mean that the proteins have evidences about their protective potential in an appropriate animal model after immunization. Other 200 non-BPA were randomly selected from the same proteomes without sharing sequence similarity to BPAs. This dataset was initially annotated with 525 features coming from 31 different annotation tools. After a feature selection step, the number of features has been reduced to 10. This short-list of 10 includes:

1. Average length of lipoprotein and other signal peptides (LipoP) (31).
2. Average length of signal peptide recognized by peptidase I (LipoP).
3. Count and length of O-beta-Glc-NAc attachment sites (YinOYang) (32).
4. Count of serine kinase specific eukaryotic phosphorylation sites (NetPhosK-S-Count) (33).
5. Average rank of human MHC alleles HLA-B matching the protein candidate (NetMhc) (34).
6. Presence of signal peptide for secretory pathway (TargetP) (35).
7. Count of I-Ag7 (MHC class II mice molecule) epitopes (GPS-MBA) (36).
8. Average score of MHC peptide binding sites (PickPocket) (37).
9. Count of scores of Furin-specific cleavage sites (ProP) (38).

The criterion to call PVC is any candidate protein with an antigen probability value greater than an a-priori threshold (0.5).

Even if the software is designed for bacterial PVCs, 8 out of 10 features are predicted by tools designed and tested for eukaryotic organisms, such as NetAcet (39) that predicts substrates of N-acetyl transferase A trained on yeast data with similar performances on mammalian substrates.

#### VacSol (21)

It is the last RV software appeared in the field of Reverse Vaccinology. It is decision-tree software that filters input protein candidates by:

1. Subcellular localization (PSORTb and CELLO2GO) (23, 40).
2. Similarity to host (human) proteins (BLASTp) (8).
3. Match to essential genes database (DEG) (41).
4. Match to virulence factors database (VFDB) (42).
5. Number of transmembrane helices (HMMTOP 2.0) (25).

The selection criterion for PVCs is: any non-host homologous, essential, virulent protein residing in the extracellular membrane with <2 transmembrane helices.

The final list of PVCs is then ranked accordingly to the prediction of MHC Class I and II binding regions and to the B-Cell epitope prediction.

## Benchmark Datasets

To compare the six RV tools, we selected a list of 11 bacterial species for which we could retrieve a list of BPAs combining information from literature (reviews) and publicly available in Protegen database (5).

The list of the 11 species -both Gram positive and Gram negative- includes bacteria that were already reported in the publications of the RV programs: eight species reported in NERVE publication or VacSol (*Neisseria gonorrhoeae, Neisseria meningitides, Staphylococcus aureus, Streptococcus pyogenes, Helicobacter pylori, Chlamydia pneumoniae, Campylobacter jejuni, Borrelia burgdorferi*), two reported in Jenner-predict publication or Vaxign (*Escherichia coli, Streptococcus pneumoniae*) and *Treponema pallidum*. For each species the list of BPAs and their relative references is reported in Table S1.

## Evaluation

Regarding Bowman-Heinson the original material has not been made available by the authors within the timelines needed to submit this manuscript. Being the pipeline of the program unavailable we decided to reproduce the analysis as far as possible in line with the description present in the articles (2).

For each bacterial species the proteome was downloaded from Uniprot database (43) version 2018_05 and was given in input to each RV program that returned in output the list of PVCs. In not specified, default settings were used for each RV program.

### Performances' Measures of RV Programs

The golden standard to measure how well a RV program performs would be in theory to purify all the pathogen's proteins, test experimentally each of them in the appropriate animal model through pathogen-specific laboratory assays and finally compare predictions and experimental results like in Table 2.

**Table 2:** Prototype of the golden-standard 2 x 2 table to measure the RV performances.

		**Immunological assays readout**	
		**Positive (BPA)**	**Negative (no BPA)**
RV method prediction	PVC	True positive (TP)	False positive (FP)
	Not-PVC	False negative (FN)	True negative (TN)

From results arranged like in Table 2 one could calculate both sensitivity or recall (TP/TP + FN), specificity (TN/TN + FP) and other performance metrics. Though in real-world scenario Table 2 is almost unfeasible because of time and cost constraints for entire bacterial proteomes that consists of thousands of proteins. In this review we decided to focus on BPAs only and accordingly to measure the performances of RV methods by:

- Fraction of proteome called PVCs (PVCs/proteome).
- Fraction of BPA identified within the set of PVCs (sensitivity or recall).
- Fold-enrichment expressed as ratio between number of BPAs observed in the set of PVCs and the number expected drawing from the proteome a random sample of the same size of the set of PVCs (statistical significance of the fold-enrichment assessed through an hypergeometric test).

## Results

### Comparison of the PVC Selection Criteria and Computational Tools

VaxiJen classifies PVCs extracting information from the chemical-physical properties of the aminoacids composing bacterial proteins. Conversely the remaining five tools in order to define PVCs work on features predicted by external programs (for a list see Table 3).

**Table 3:** Summary of the external computational programs used by the six programs to predict the protein features instrumental to filter or classify PVCs.

**Protein feature**	**Prediction program**	**NERVE**	**Vaxign**	**Jenner-predict**	**VacSol**	**Bowman-Heinson**
Subcellular localization	Psortb	X	X	X	X	
	TargetP					X
Transmembrane domains	HMMTOP	X	X	X	X	
Pathogenic domains or virulent factors	SPAAN	X	X			
	Pfam			X		
	VFDB				X	
	LipoP					X
Similarity to host proteins	BLASTp against MHCPEP db	X				
	BLASTp against RefSeq and Swiss Prot db				X	
	OrthoMCL		X			
B-T cell response	NetMhc					X
	Vaxitope		X			
	ABCPred				X	
	ProPred-I				X	
	ProPred				X	
	GPS-MBA					X
	PickPocket					X
Post-translational modification	YinOYang (glycosylation)					X
	NetPhosK (phosphorylation)					X
	ProP (proprotein convertase cleavage)					X

From the comparison of the PVCs selecting criteria of these five RV programs we observed that they share two common features:

1. Extracellular subcellular localization
2. Probability of being a virulence factor

About the prediction of the extracellular localization the RV programs use mostly Psort while Bowman-Heinson implements TargetP.

The major virulence characteristic that is searched for is adhesion. SPAAN is the software of election to predict the probability of a protein being an adhesin and is used by NERVE and Vaxign. VacSol searches PVCs in the database of virulence factors VFDB that contains discrete proportion of adhesins. Among the Pfam domains used by Jenner-predict 96 domains are reported as related to adhesion. Also lipoproteins have been shown to play key roles in adhesion to host cells and translocation of virulence factors into host cells (44). Heinson uses LipoP software that produces predictions of lipoproteins.

Differently from what one might expect not all the RV programs use the sequence similarity to host proteins (either mouse or human) as a selecting criterion. Jenner-predict for instance uses the homology to human proteins only to rank the PVCs accordingly to what they call their “vaccine potential.” Machine-learning approach of Heinson doesn't include in the list of the 525 initial potential discriminative features anything related to homology or similarity to host proteins.

Finally, HMMTOP is common to all the four decision-tree programs NERVE, Vaxign, Jenner-predict and VacSol. It is used to predict the number of transmembrane domains that is directly linked to the likelihood each protein has to be successfully purified.

### Running Time

The performances in terms of time needed to predict PVCs are reported in Table 4. Time has been calculated using a set of 100 protein sequences with an average length of 360 aminoacids. Tools like Vaxign and VaxiJen are very fast and are able to predict 100 proteins in a few seconds or minutes, instead other tools like NERVE, Bowman-Heinson and VacSol are slower and need between 15 and 60 min to analyze the same protein dataset on a MacBook Pro (2.6 GHz Intel Core i7, 16 Gb RAM).

**Table 4:** Summary of run times on a benchmark dataset of 100 proteins (average length 360 a.a.).

**Program**	**Running time**
VaxiJen	5 s
Vaxign	5 min 40 s
NERVE	17 min 37 s
Bowman-Heinson	27 min 8 s
VacSol	49 min 40 s

This difference is due to the fact that tools used via browser like Vaxign and VaxiJen have been developed in a specific way integrating the software needs with the hardware. In the case of tools such as NERVE, VacSol and Bowman-Heinson, the analysis depends on the characteristics of the hardware used and the running time may vary depending on the capabilities of the system.

In addition, must be noticed that tools like NERVE, VacSol and Bowman-Heinson are not available as preconfigured virtual machine so time must be dedicated to install the software itself and all its dependencies. Vaxign and VaxiJen, available via browser, are easier to use, only necessitating to copy and paste fasta sequences of the proteins.

### Fraction of PVCs

The results are presented in Table 5 where pathogens are listed following the order of their proteome size (decreasing order).

**Table 5:** Fraction of PVCs predicted by each of the six programs (NERVE, VaxiJen, Vaxign, VacSol, Bowman-Heinson, and Jenner-predict) where pathogens are listed following the order of their proteome size.

**Species**	**RV programs**					
	**NERVE**	**VaxiJen**	**Vaxign**	**VacSol**	**Bowman-Heinson**	**Jenner-predict**
*Escherichia coli*	627 (11.7%)	1,979 (37%)	452 (8.5%)	174 (3.3%)	661 (12.4%)	250 (4.7%)
*Streptococcus pyogenes*	690 (19.2%)	972 (27%)	504 (14%)	190 (5.3%)	414 (11.5%)	121 (3.4%)
*Chlamydia pneumoniae*	398 (11.8%)	984 (29.2%)	330 (9.8%)	25 (0.7%)	380 (11.3%)	260 (7.7%)
*Staphylococcus aureus*	254 (9.5%)	992 (37.3%)	125 (4.7%)	118 (4.4%)	300 (11.3%)	126 (4.7%)
*Streptococcus pneumoniae*	194 (9.2%)	625 (29.6%)	122 (5.8%)	111 (5.3%)	216 (10.2%)	75 (3.6%)
*Neisseria gonorrhoeae*	272 (12.9%)	917 (43.5%)	197 (9.3%)	45 (2.1%)	304 (14.4%)	81 (3.8%)
*Neisseria meningitidis*	256 (12.8%)	815 (40.7%)	180 (9%)	37 (1.8%)	308 (15.4%)	88 (4.4%)
*Treponema pallidum*	92 (5.6%)	682 (41.3%)	85 (5.2%)	20 (1.2%)	234 (14.2%)	58 (3.5%)
*Campylobacter jejuni*	199 (12.2%)	530 (32.6%)	111 (6.8%)	39 (2.4%)	211 (13%)	60 (3.7%)
*Helicobacter pylori*	201 (13.5%)	429 (28.7%)	131 (8.8%)	40 (2.7%)	231 (15.5%)	81 (5.4%)
*Borrelia burgdorferi*	213 (16.5%)	432 (33.5%)	96 (7.4%)	10 (0.8%)	186 (14.4%)	25 (1.9%)
Average on 27,247 total proteins	3,396 (12.5%)	9,357 (34.3%)	2,602 (9.5%)	809 (3%)	3,445 (12.6%)	1,225 (4.5%)

**Figure 2:**
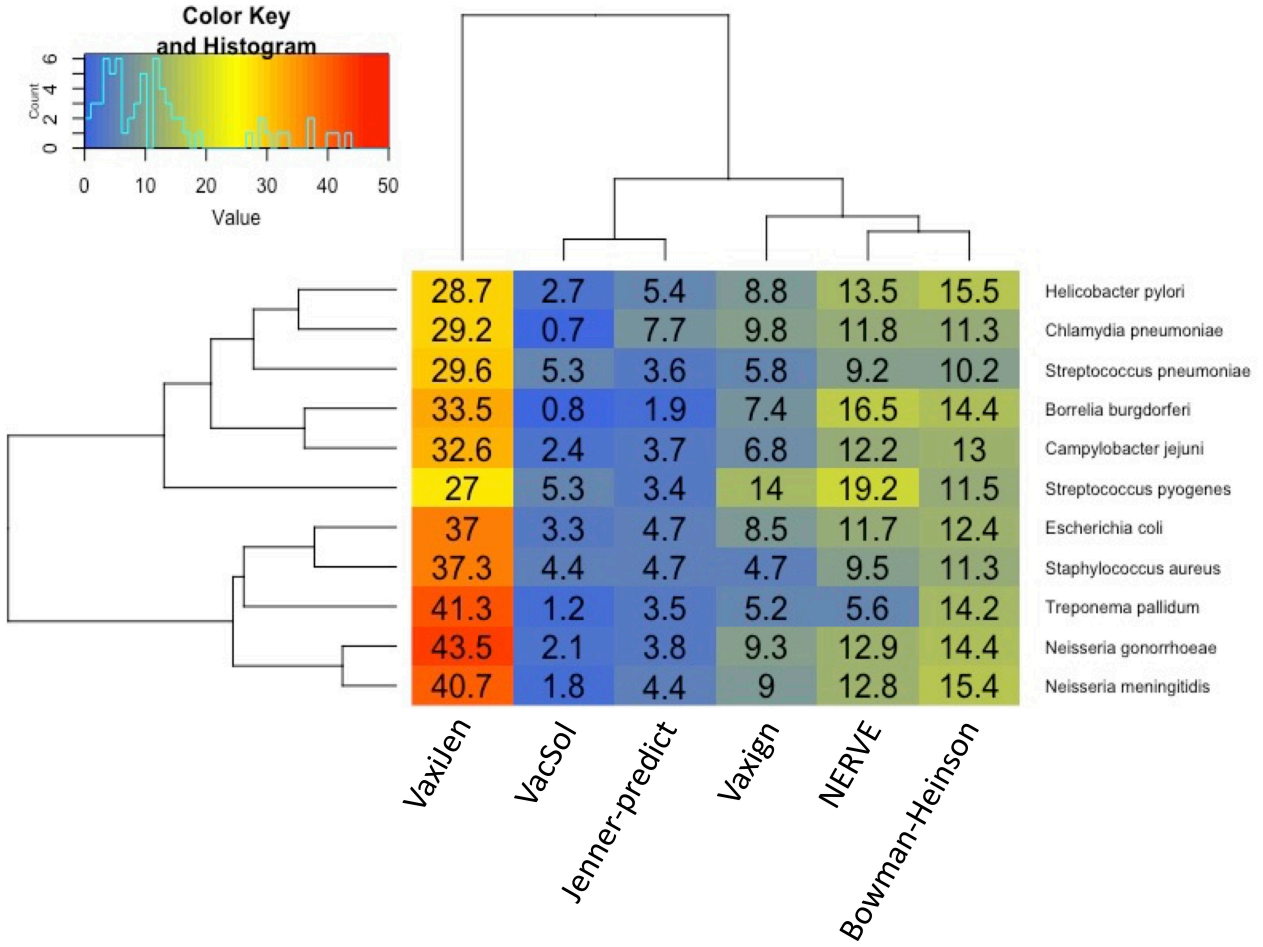
Hierarchical clustering of RV programs based on the fraction (%) of proteome predicted as PVCs. Columns correspond to the six programs and rows to the 11 pathogens' proteomes. Legend shows the color code of fraction of proteome predicted as PVCs.

Among the six programs VacSol resulted to be the most conservative predicting as PVCs on average only 3% of a bacterial proteome (min 0.7% *Chlamydia pneumoniae*—max 5.3% *Streptococcus pyogenes*). On the opposite side VaxiJen is the most permissive with on average 34.4% (min 27% *Staphylococcus aureus*—max 43.5% *Neisseria gonorrhoeae*) of a bacterial proteome predicted as PVC. A graphical summary is provided in Figure 2. As shown in the figure based on proteome fraction predicted as PVC we could hierarchically cluster the six programs into three groups corresponding to high, medium and low fraction of predicted PVCs. VaxiJen is the software that predicts the greatest fractions of PVCs (always more than 25% of a proteome) and stands separately from the other tools. VacSol and Jenner-predict constitute the second group with low fractions of PVCs (always <10% of a proteome). In the middle are NERVE, Vaxign and Bowman-Heinson with similar medium fractions of PVCs predicted.

**Figure 3:**
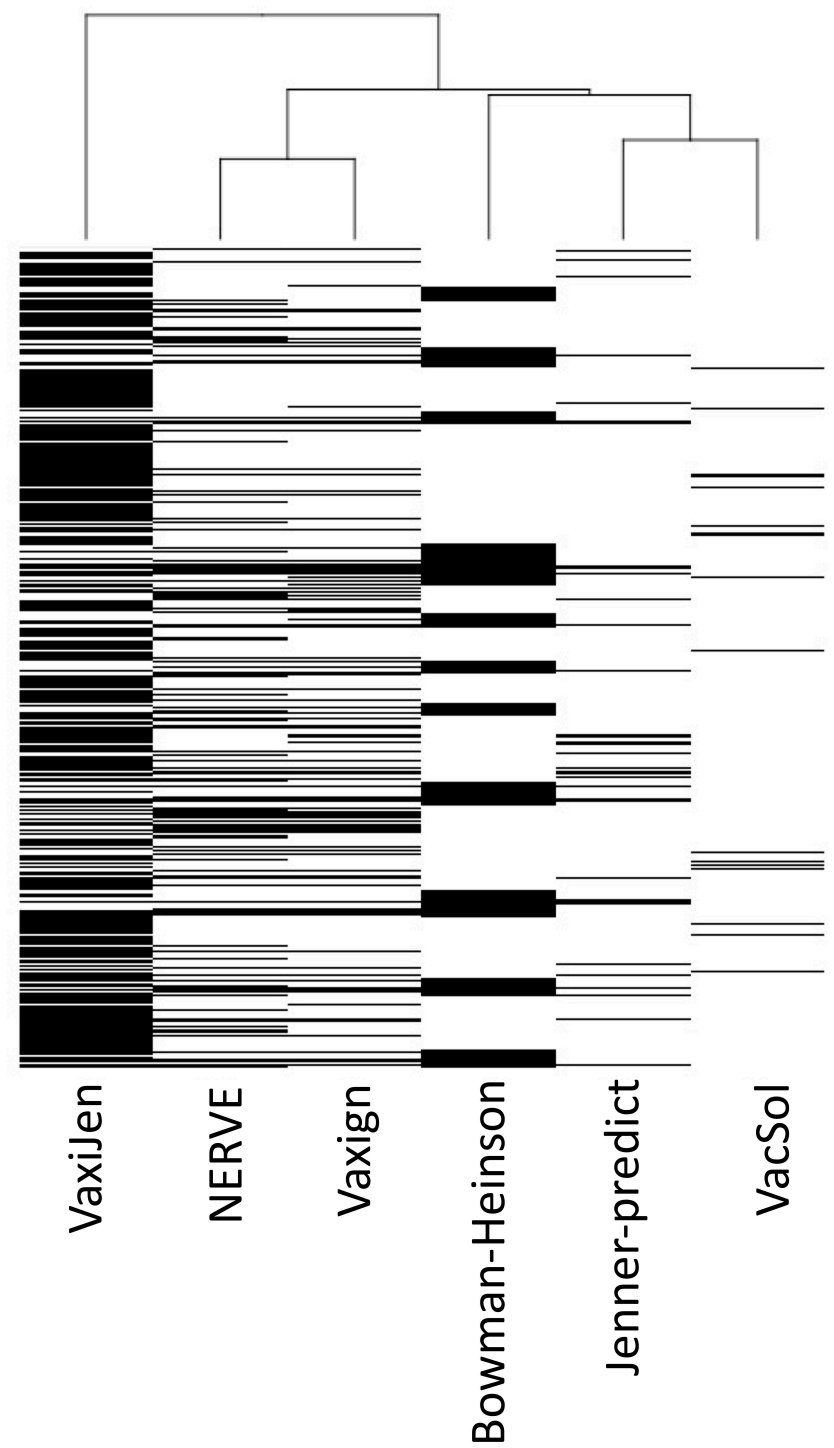
Hierarchical clustering of the six RV programs (columns) based on PVC calls for 27,247 total proteins (rows). Each cell corresponds to the output of each program for each protein: white colored means not-PVC, black colored means PVC.

Analyzing the output of the six RV programs for each single protein we observed heterogeneous agreement among the programs (Figure 3). To quantify the strength of each pair-wise agreement among the six programs we used the Choen's kappa (45). If two programs are in complete agreement, then kappa is equal to 1. If there is no agreement between two programs other than what would be expected by chance kappa is equal or even <0. The values of kappa for the pairwise comparisons between programs are given in Table 6.

**Table 6:** Choen's kappa values for the pair-wise agreement between programs.

	**VacSol**	**VaxiJen**	**Vaxign**	**NERVE**	**Bowman-Heinson**
VaxiJen	−0.012				
Vaxign	−0.016	0.129			
NERVE	−0.014	0.135	**0.769**		
Bowman-Heinson	0.009	0.126	0.278	0.276	
Jenner-predict	0.032	0.073	0.321	0.269	0.275

*In bold the maximum value of each column*.

The programs are scarcely in agreement with the only exception of NERVE and Vaxign that show a high kappa value (0.769). VacSol seems to be the software that returns a list of PVCs mostly not in common with others (kappa ranges between −0.012 and 0.032).

### Fraction of BPAs Identified and Fold-Enrichment

For each software we measured the fraction of BPAs identified in the subset of PVCs, the recall and the fold-enrichment associated with *p*-value based on hypergeometric distribution as described in section Performances' Measures of RV Programs.

As reported in Table 7 the software with the highest fold enrichment is Jenner-predict that however has a recall of 44%. VaxiJen recalls the maximum absolute number of BPAs (76 BPAs in 9,357 PVCs) but has a low fold-enrichment (2.2). In comparison to VaxiJen, Bowman- Heinson with 3,445 PVCs recalls 75 BPAs showing therefore the best performance in terms of combined recall and fold-enrichment (5.9). Data for each single pathogen are provided in Table S2.

**Table 7:** Summary of the performance on the RV programs in terms of recall of BPAs and fold-enrichment.

**Software**	**PVCs**	**Observed BPAs**	**Recall (%)**	**Expected BPAs**	**Fold-enrichment**	***p*-Value**
NERVE	3,396	64	64	12	5.1	1.51E-33
VaxiJen	9,357	**76**	**76**	**34**	2.2	1.80E-17
Vaxign	2,602	58	58	10	6.1	1.90E-33
VacSol	809	4	4	3	1.3	3.46E-01
Bowman-Heinson	3,445	75	75	13	5.9	1.99E-46
Jenner-predict	1,225	44	44	4	**9.8**	1.09E-32

*In bold the maximum value of each column. Numbers are referred to the total number of proteins (27,247) of the 11 pathogens. BPAs are 100 in total*.

## Discussion

Reverse vaccinology represents a critical step toward the discovery and development of protein subunit vaccines.

From its conception in early 2000 to date several programs have been developed to do Reverse Vaccinology. We reviewed six of them, open-source, designed for bacterial pathogens.

We found two types of RV programs: those based on decision-tree or filtering and those based on machine-learning or classifying.

The first type—including NERVE, Jenner-predict, Vaxign, and VacSol—has the advantage of using a predefined set of core features to predict PVCs, without requiring training on a preexisting list of good and bad candidates. Core features include extracellular localization, probability to be an adhesion, lack of similarity to host proteins and limited number of transmembrane domains.

We observed that on average 10–15% of a bacterial proteome matches these criteria, resulting in a list of hundreds of proteins to be potentially tested in preclinical laboratories.

Conversely, methods based on machine-learning use training sets. VaxiJen uses as predictive features values calculated from the aminoacidic composition of the proteins and returns long lists of PVCs: on average one third of a bacterial proteome is called PVC. It is likely that changing the training set—at the time of writing the review composed by 200 proteins—the output lists of PVCs might change as well. The other machine-learning approach (based on a Support Vector Machine) developed by Bowman and enhanced by Heinson uses features extracted from programs predictive of subcellular localization, B and T cell responses and post-translational modifications. Differently by VaxiJen the output list of PVCs is contained (12% of a proteome on average) and the method shows in our benchmark dataset a valuable enrichment in BPAs.

One advantage of the filtering RV programs is represented by user's full control of the step-wise process toward the selection of PVCs. PVCs are then easy to interpret and communicate. NERVE has not been updated since its release though Vaxign constitutes a valid alternative as it implements a very similar pipeline. The accordance between the two is indeed very good. VacSol represents also a valid RV filtering program but the number of resulting PVCs is so restricted that the likelihood to miss good candidates is not negligible.

Machine-learning methods are able to rank all the proteins of a pathogen based on their likelihood to be a PVCs. They can handle simultaneously much more features than filtering RV methods. However, these methods need an *a-priori* training dataset of good and bad antigens. This represents their main Achille's heel because if it true that on literature one might found experimental evidences for good antigens, the same is not always valid for negative cases i.e., candidate proteins that didn't succeed in preclinical testing. The shortcut commonly used to artificially populate a set of bad antigens randomly selecting proteins not tested in laboratory but with scarce similarity to good antigens is questionable. Evidence of this are for instance the two antigens fHbp and NadA present in *Bexsero*® vaccine. Considering fHbp a good antigen, based on the almost null sequence similarity to NadA one would consider NadA as bad candidate. It would be beneficial to increase the performances of RV methods if manually curated set of candidate proteins with negative experimental outcomes would be publicly available. A limitation of machine-learning RV methods might be represented by the interpretation of the results since it is not straightforward to map backwardly PVCs to the features space.

## Conclusions

We have extensively reviewed, for the first time, the state-of-the-art of Reverse Vaccinology bioinformatic tools used in bacterial antigen prioritization, visualized their diversity, and examined their performances.

We found that independently by the number of predicted PVCs, none of the six programs was able to recall more than 76 BPAs out of the benchmark list of 100 composed from eleven different bacterial species. The machine learning based method of Bowman-Heinson demonstrated the best ratio between BPA identified and number of PVCs predicted, recalling 75% of BPAs in a total of 3,445 PVCs. This is relevant in the filed because while reducing the number of laboratory tests this method should simultaneously guarantee the identification of the vast majority of proteins with potential protective efficacy.

When we looked at the overall agreement in terms of PVC calls among the six programs we found a low score indicating that each program capture a specific profile for PVCs. Being the time of processing reasonable we suggest to explore the results of at least one filtering and one classifying method. We finally observed that a distinguishing feature in the most cited and applied RV packages VaxiJen and Vaxign, is their accessibility to final users through graphical user interfaces. We encourage researches in this field to invest in the development of user-friendly interfaces, as much as to the improvement of the predictive power of the algorithms.

## Author Contributions

All authors contributed to methods design, editing, and approved the final manuscript. AB and MD wrote and tested code, sourced data, performed data analysis, and drafted the manuscript. All authors read and approved the final manuscript.

### Conflict of Interest Statement

AB, DM, and RR were employees of GSK group of companies at the time of the study. MD is a Ph.D. student at the University of Turin and participates in a postgraduate studentship program at GSK.

## Abbreviations

RV: reverse vaccinology
MenB: Neisseria meningitidis serogroup B, Meningococcus B
PVC: potential vaccine candidate
VC: vaccine candidate
VT: vaccine target
BPA: bacterial protective antigen
PAg: protective antigen
KA: known antigen
KPA: known protective antigen
NERVE: new enhanced reverse vaccinology environment
BLAST: basic local alignment search tool
PSI-BLAST: position-specific iterated basic local alignment search tool
SBA: serum bactericidal assay
DEG: database of essential genes
OMV: outer membrane vesicle
ACC: auto cross covariance
SVM: support vector machine
MHC: major histocompatibility complex
MHC: major histocompatibility complex class I
MHC: major histocompatibility complex class II
VFDB: virulence factor database
TP: true positive
TN: true negative
FP: false positive
FN: false negative
fHbp: meningococcal factor H binding protein
NadA: Neisseria adhesin A.
