# Supplementary material for: Comparison of Open-Source Reverse Vaccinology Programs for Bacterial Vaccine Antigen Discovery
Source: Front Immunol. 2019 Feb 14;10:113. doi: 10.3389/fimmu.2019.00113 (PMC6382693; doi:10.3389/fimmu.2019.00113)
Supplement: Supplementary file 1 [file Table_1.docx]

**Table S1:** Descriptive information for the 100 bacterial protective antigens (BPAs) used in this study to calculate the recall and fold-enrichment.

| **Pathogen** | **Strain** | **Number of known bacterial antigens** | **Uniprot ID of known bacterial antigens** | **References** |
| --- | --- | --- | --- | --- |
| *Neisseria gonorrhoeae* | ATCC 700825/ FA 1090 | 10 | AniA (Q5F7A4_NEIG1)  TbpA (Q5F574_NEIG1)  TbpB (Q5F6Q3_NEIG1)  MtrE (Q5F726_NEIG1)  PorB (Q5F5V7_NEIG1)  Ompa (Q5F6J5_NEIG1)  LbpA (Q5F6Q4_NEIG1)  TdfJ (Q5F7H3_NEIG1)  Lst (Q5F7T9_NEIG1)  Nspa (Q5FA01_NEIG1) | Vivona S. et al., 2006  Edwards J. et al., 2016 |
| *Staphylococcus aureus* | MW2 | 12 | ClfA (CLFA_STAAW)  MntC (A0A0H3JW04_STAAW)  IsdB (ISDB_STAAW)  ClfB (CLFB_STAAW)  fhuD2 (A0A0H3K1A0_STAAW)  EsxA (ESXA_STAAW)  EsxB (ESXB_STAAW)  Hla (A0A0H3K2R6_STAAW)  Sta011 (Y071_STAAW)  Spa (A0A0H3K1I2_STAAW)  Cna (A0A0H3JZE1_STAAW)  mecA (A0A0H3JUR9_STAAW) | Yang et al., 2011  Giersing et al., 2016  Yeaman et al., 2014 |
| *Streptococcus pyogenes* | M1 | 15 | spy0416 (Q9A180_STRP1)  spy0167 (TACY_STRP1)  spy0269 (Q9A1H3_STRP1)  spy0469 (J7M2V9_STRP1)  spy1228 (Q99ZH4_STRP1)  spy1801 (Q99Y99_STRP1)  SpeB (J7M934_STRP1)  SpeA (J7M5V3_STRP1)  SpeC (SPEC_STRP1)  Cpa (J7M2J0_STRP1)  SPy_0128 (PILIN_STRP1)  SPy_0130 (Q9A1S0_STRP1)  cell surface protein (J7MBD1_STRP1)  M protein (Q99XV0_STRP1)  Mac (Q7DAM2_STRP1) | Yang B. et al., 2011  Mortensen R. et al., 2016  Steer A. et al., 2016  Bensi G. et al., 2012 |
| *Helicobacter pylori* | J99 / ATCC 700824 | 11 | Catalase (CATA_HELPJ)  GltA (CISY_HELPJ)  Hsp60 (CH60_HELPJ)  vacA (VACA_HELPJ)  HspA (CH10_HELPJ)  NAP (DPS_HELPJ)  oipA (Q9ZLJ8_HELPJ)  ureB (URE1_HELPJ)  cagA (CAGA_HELPJ)  HpaA (HPAA_HELPJ)  SOD (SODF_HELPJ) | Yang B. et al., 2011  Mirzaei N. et al., 2017  Naz A. et al., 2015 |
| *Escherichia coli* | CFT073 / ATCC 700928 / UPEC | 10 | ybcU (A0A0H2V6N9_ECOL6)  c2436 (A0A0H2V8C1_ECOL6)  c2482 (A0A0H2V8J2_ECOL6)  c5174 (A0A0H2VDP0_ECOL6)  iutA (A0A0H2VAM3_ECOL6)  fimH (A0A0H2VDU7_ECOL6)  iroN (A0A0H2V663_ECOL6)  c0393 (A0A0H2V4L3_ECOL6)  upaG (UPAG_ECOL6)  c3389 (A0A0H2VA37_ECOL6) | Yang B. et al., 2011  Svennerholm A. M., Tobias J., 2008  Zhang H., 2017  Bourgeois A. L., 2016 |
| *Chlamydia pneumoniae* | ATCC VR-2282 | 7 | pmp10 (PMP10_CHLPN)  pmp2 (PMP2_CHLPN)  ArtJ (ARTJ_CHLPN)  Eno (ENO_CHLPN)  HtrA (A0A0F7WNW8_CHLPN)  OmpH (SKPL_CHLPN)  MomP (MOMP_CHLPN) | Vivona S. et al., 2006  Capo S., 2005  Finco O., 2005 |
| *Campylobacter jejuni* | ATCC 700819 / NCTC 11168 | 7 | CadF (Q0P8D9_CAMJE)  CjaA (Q0P9S0_CAMJE)  FlaA (FLA1_CAMJE)  FlaC (FLAC_CAMJE)  FspA1 (Q0PA31_CAMJE)  Peb1A (PEB1A_CAMJE)  PorA (PORA_CAMJE) | Yang B. et al., 2011  O’Ryan M., 2015  Baqar S., 2008  Lee L. H., 1999  Buckley A. M., 2010 |
| *Borrelia burgdorferi* | ATCC 35210 / B31 / CIP 102532 / DSM 4680 | 6 | OspA (OSPA_BORBU)  OspC (OSPC_BORBU)  DbpA (DBPA_BORBU)  OspB (OSPB_BORBU)  BBK32 (O50835_BORBU)  Fla (FLA1_BORBU) | Yang B. et al., 2011  Schuijt T. J., 2011 |
| *Treponema pallidum* | Nichols | 4 | GlpQ (GLPQ_TREPA)  TmpB (TMPB_TREPA)  Tp92 (TP326_TREPA)  Tp0821 (R9UVI4_TREPA) | Yang B. et al., 2011  Kubanov A., 2017  Lithgow K.V., Cameron C.E., 2017 |
| *Streptococcus pneumoniae* | TIGR4 | 14 | ClpP (CLPP_STRPN)  GltX (SYE_STRPN)  Gnd (A0A0H2UNG6_STRPN)  LplA (A0A0H2UQ30_STRPN)  DnaJ (DNAJ_STRPN)  PsaA (MTSA_STRPN)  Ply (TACY_STRPN)  CbpA (A0A0H2US50_STRPN)  PcpA (A0A0H2USF9_STRPN)  PspA (A0A0H2UMZ8_STRPN)  SP0148 (A0A0H2UN58_STRPN)  SP1912 (A0A0H2URM0_STRPN)  SP2108 (MALX_STRPN)  A0A0H2UN78_STRPN | Yang B. et al., 2011  Entwisle C., 2017  Qiu Y., 2017 |
| *Neisseria meningitidis* | MC58 | 4 | Fhbp (Q9JXV4_NEIMB)  NadA (Q9JXK7_NEIMB)  NHBA (Q7DD37_NEIMB)  porA (OMPA_NEIMB) | Serruto D., 2012  Gorringe A. R., 2012 |

**Bibliography**

Baqar, S., Applebee, L. A., Gilliland, T. C., Lee, L. H., Porter, C. K., and Guerry, P. (2008). Immunogenicity and protective efficacy of recombinant Campylobacter jejuni flagellum-secreted proteins in mice. Infect. Immun. 76, 3170–3175. doi:10.1128/IAI.00076-08.

Bensi, G., Mora, M., Tuscano, G., Biagini, M., Chiarot, E., Bombaci, M., et al. (2012). Multi High-Throughput Approach for Highly Selective Identification of Vaccine Candidates: the Group A Streptococcus Case. Mol. Cell. Proteomics 11, M111.015693. doi:10.1074/mcp.M111.015693.

Bourgeois, A. L., Wierzba, T. F., and Walker, R. I. (2016a). Status of vaccine research and d evelopment for enterotoxigenic Escherichia coli. Vaccine 34, 2880–2886. doi:10.1016/j.vaccine.2016.02.076.

Bourgeois, A. L., Wierzba, T. F., and Walker, R. I. (2016b). Status of vaccine research and development for enterotoxigenic Escherichia coli. Vaccine 34, 2880–2886. doi:10.1016/j.vaccine.2016.02.076.

Buckley, A. M., Wang, J., Hudson, D. L., Grant, A. J., Jones, M. A., Maskell, D. J., et al. (2010). Evaluation of live-attenuated Salmonella vaccines expressing Campylobacter antigens for control of C. jejuni in poultry. Vaccine 28, 1094–1105. doi:10.1016/j.vaccine.2009.10.018.

Edwards, J. L., Jennings, M. P., Apicella, M. A., and Seib, K. L. (2016a). Is gonococcal disease preventable? The importance of understanding immunity and pathogenesis in vaccine development. Crit. Rev. Microbiol. 42, 928–941. doi:10.3109/1040841X.2015.1105782.

Edwards, J. L., Jennings, M. P., Apicella, M. A., and Seib, K. L. (2016b). Is gonococcal disease preventable? The importance of understanding immunity and pathogenesis in vaccine development. Crit. Rev. Microbiol. 42, 928–941. doi:10.3109/1040841X.2015.1105782.

Entwisle, C., Hill, S., Pang, Y., Joachim, M., McIlgorm, A., Colaco, C., et al. (2017). Safety and immunogenicity of a novel multiple antigen pneumococcal vaccine in adults: A Phase 1 randomised clinical trial. Vaccine 35, 7181–7186. doi:10.1016/j.vaccine.2017.10.076.

Finco, O., Bonci, A., Agnusdei, M., Scarselli, M., Petracca, R., Norais, N., et al. (2005). Identification of new potential vaccine candidates against Chlamydia pneumoniae by multiple screenings. Vaccine 23, 1178–1188. doi:10.1016/j.vaccine.2004.07.045.

Giersing, B. K., Dastgheyb, S. S., Modjarrad, K., and Moorthy, V. (2016). Status of vaccine research and development of vaccines for Staphylococcus aureus. Vaccine 34, 2962–2966. doi:10.1016/j.vaccine.2016.03.110.

Gorringe, A. R., and Pajon, R. (2012a). Bexsero: A multicomponent vaccine for prevention of meningococcal disease. Hum. Vaccines Immunother. 8, 164–173. doi:10.4161/hv.18500.

Gorringe, A. R., and Pajon, R. (2012b). Bexsero: A multicomponent vaccine for prevention of meningococcal disease. Hum. Vaccines Immunother. 8, 164–173. doi:10.4161/hv.18500.

Kubanov, A., Runina, A., and Deryabin, D. (2017). Novel Treponema pallidum Recombinant Antigens for Syphilis Diagnostics: Current Status and Future Prospects. Biomed Res. Int. 2017. doi:10.1155/2017/1436080.

Lee, L. H., Burg, E., Baqar, S., Bourgeois, A. L., Burr, D. H., Ewing, C. P., et al. (1999). Evaluation of a truncated recombinant flagellin subunit vaccine against Campyiobacter jejuni. Infect. Immun. 67, 5799–5805. Available at: https://www.ncbi.nlm.nih.gov/pmc/articles/PMC96957/pdf/ii005799.pdf [Accessed April 17, 2018].

Mirzaei, N., Poursina, F., Moghim, S., Rashidi, N., and Ghasemian Safaei, H. (2017). The study of H. pylori putative candidate factors for single- and multi-component vaccine development. Crit. Rev. Microbiol. 43, 631–650. doi:10.1080/1040841X.2017.1291578.

Mortensen, R., Nissen, T. N., Fredslund, S., Rosenkrands, I., Christensen, J. P., Andersen, P., et al. (2016a). Identifying protective Streptococcus pyogenes vaccine antigens recognized by both B and T cells in human adults and children. Sci. Rep. 6, 1–11. doi:10.1038/srep22030.

Mortensen, R., Nissen, T. N., Fredslund, S., Rosenkrands, I., Christensen, J. P., Andersen, P., et al. (2016b). Identifying protective Streptococcus pyogenes vaccine antigens recognized by both B and T cells in human adults and children. Sci. Rep. 6. doi:10.1038/srep22030.

Naz, A., Awan, F. M., Obaid, A., Muhammad, S. A., Paracha, R. Z., Ahmad, J., et al. (2015). Identification of putative vaccine candidates against Helicobacter pylori exploiting exoproteome and secretome: A reverse vaccinology based approach. Infect. Genet. Evol. 32, 280–291. doi:10.1016/j.meegid.2015.03.027.

O’Ryan, M., Vidal, R., Del Canto, F., Salazar, J. C., and Montero, D. (2015). Vaccines for viral and bacterial pathogens causing acute gastroenteritis: Part II: Vaccines for Shigella, Salmonella, enterotoxigenic E. Coli (ETEC) enterohemorragic E. Coli (EHEC) and Campylobacter jejuni. Hum. Vaccines Immunother. 11, 601–619. doi:10.1080/21645515.2015.1011578.

Qiu, Y., Zhang, X., Wang, H., Zhang, X., Mo, Y., Sun, X., et al. (2017). Heterologous prime-boost immunization with live SPY1 and DnaJ protein of Streptococcus pneumoniae induces strong Th1 and Th17 cellular immune responses in mice. J. Microbiol. 55, 823–829. doi:10.1007/s12275-017-7262-1.

Schuijt, T. J., Hovius, J. W., van der Poll, T., van Dam, A. P., and Fikrig, E. (2011a). Lyme borreliosis vaccination: The facts, the challenge, the future. Trends Parasitol. 27, 40–47. doi:10.1016/j.pt.2010.06.006.

Schuijt, T. J., Hovius, J. W., van der Poll, T., van Dam, A. P., and Fikrig, E. (2011b). Lyme borreliosis vaccination: The facts, the challenge, the future. Trends Parasitol. 27, 40–47. doi:10.1016/j.pt.2010.06.006.

Svennerholm, A. M., and Tobias, J. (2008). Vaccines against enterotoxigenic Escherichia coli. Expert Rev. Vaccines 7, 795–804. doi:10.1586/14760584.7.6.795.

Yeaman, M. R., Filler, S. G., Schmidt, C. S., Ibrahim, A. S., Edwards, J. E., and Hennessey, J. P. (2014). Applying convergent immunity to innovative vaccines targeting Staphylococcus aureus. Front. Immunol. 5, 1–21. doi:10.3389/fimmu.2014.00463.

Zhang, H., Xu, Y., Zhang, Z., You, J., Yang, Y., and Li, X. (2017). Protective immunity of a Multivalent Vaccine Candidate against piglet diarrhea caused by enterotoxigenic Escherichia coli (ETEC) in a pig model. Vaccine 36, 723–728. doi:10.1016/j.vaccine.2017.12.026.
